# Hematopoietic Stem Cell Transplantation for Patients with Paroxysmal Nocturnal Hemoglobinuria with or without Aplastic Anemia: A Multicenter Turkish Experience

**DOI:** 10.4274/tjh.galenos.2021.2021.0105

**Published:** 2021-08-25

**Authors:** Fergün Yılmaz, Nur Soyer, Güldane Cengiz Seval, Sinem Civriz Bozdağ, Pervin Topçuoğlu, Ali Ünal, Leylagül Kaynar, Gökhan Özgür, Gülsan Sucak, Hakan Göker, Mustafa Velet, Hakan Özdoğu, Mehmet Yılmaz, Emin Kaya, Ozan Salim, Burak Deveci, İhsan Karadoğan, Güray Saydam, Fahri Şahin, Filiz Vural

**Affiliations:** 1 Marmara University Faculty of Medicine, Department of Hematology, İstanbul, Turkey; 2 Ege University Faculty of Medicine, Department of Hematology, İzmir, Turkey; 3 Ankara University Faculty of Medicine, Department of Hematology, Ankara, Turkey; 4 Erciyes University Faculty of Medicine, Department of Hematology, Ankara, Turkey; 5 Medical Park Bahçeşehir Hospital, Clinic of Hematology and Transplantation, İstanbul, Turkey; 6 Hacettepe University Faculty of Medicine, Department of Hematology, Ankara, Turkey; 7 Başkent University Faculty of Medicine, Adana Bone Marrow Transplantation Center, Department of Hematology, Adana, Turkey; 8 SANKO University Faculty of Medicine, Department of Hematology, Gaziantep, Turkey; 9 İnönü University Faculty of Medicine, Department of Hematology, Malatya, Turkey; 10 Akdeniz University Faculty of Medicine, Department of Hematology, Antalya, Turkey; 11 İstanbul Gelişim University, Medstar Antalya Hospital Bone Marrow Transplantation Center, Department of Hematology, Antalya, Turkey

**Keywords:** Paroxysmal nocturnal hemoglobinuria, Transplantation, Allogeneic stem cell transplantation, Aplastic anemia

## Abstract

**Objective:**

Although inhibition of the complement system at different steps is a promising therapy modality in patients with paroxysmal nocturnal hemoglobinuria (PNH), allogeneic hematopoietic stem cell transplantation (HCT) is still the only curative therapy, especially for patients with intractable hemolysis or bone marrow failure. The aim of this study is to evaluate the outcomes of allogeneic HCT in PNH patients with aplastic anemia (PNH-AA) or without.

**Materials and Methods:**

Thirty-five PNH/PNH-AA patients who were treated with allogeneic HCT in 10 transplantation centers in Turkey were retrospectively analyzed.

**Results:**

Sixteen (45.7%) and 19 (54.3%) patients were diagnosed with classical PNH and PNH-AA, respectively. The median age of the patients was 32 (18-51) years. The 2-year overall survival (OS) rate and rate of graft-versus-host disease-free, failure-free survival (GFFS) was 81.2% and 78.1%, respectively. The 2-year OS in cases of classical PNH and PNH-AA was 81.3% and 79.9%, respectively (p=0.87), and 2-year GFFS in cases of PNH and PNH-AA was 79% and 76% (p=0.977), without statistical significance. The OS and GFFS rates also did not differ between transplantations with matched sibling donors (MSDs) and matched unrelated donors (MUDs).

**Conclusion:**

Allogeneic HCT with MSDs or MUDs is a good option for selected patients with classical PNH and PNH-AA. In particular, patients with debilitating and refractory hemolysis and patients with bone marrow failure might form an excellent group of candidates for allogeneic HCT.

## Abstract (tr)

**Amaç:**

Paroksismal noktürnal hemoglobunüri (PNH) hastalarının tedavisinde kompleman sisteminin değişik basamaklardaki inhibisyonu umut vadedici bir tedavi yöntemidir. Ancak hastalığın belirti ve bulgularını kontrol etmekte etkin bir tedavi yöntemi olsa da küratif bir tedavi seçeneği değildir. Özellikle kompleman inhibisyonuna rağmen devam eden hemolizi, replasman ihtiyacı ve kemik iliği yetmezliği ile birlikteliği olan hasta grubunda halen tek küratif tedavi seçeneği allojeneik kök hücre naklidir (KHN). Bu çalışmamızda aplastik anemi ile birlikteliği olan (PNH-AA) ve olmayan PNH hastalarında yapılmış allojeneik KHN’nin sonuçlarını değerlendirmeyi amaçladık.

**Gereç ve Yöntemler:**

On ayrı merkezde PNH/PNH-AA tanısı olup allojeneik KHN yapılan toplam 35 hasta retrospektif olarak tarandı.

**Bulgular:**

On altı (%45,7) PNH ve 19 (%54,3) PNH-AA hastası çalışmaya dahil edildi. Hastaların ortanca yaşı 32 (18-51) idi. Hastalardaki sağkalım analizlerine baktığımızda; 2 yıllık genel sağkalım (GS) ve graft versus host hastalıksız ve graft yetmezliksiz sağkalım (GFFS) oranları sırasıyla %81,2 ve %78,1 idi. İki yıllık sağkalım PNH ve PNH-AA hastalarında sırasıyla %81,3 ve %79,9 olarak hesaplandı ve gruplar arasında istatistiksel bir fark tespit edilmedi (p=0,87). Tam uyumlu akraba donör ve tam uyumlu akraba dışı donör alt grupları karşılaştırıldığında 2 yıllık OS ve GFFS oranlarında istatistiksel fark gözlenmedi.

**Sonuç:**

Seçilmiş PNH ve PNH-AA hasta grubunda akraba veya akraba dışı tam uyumlu donör ile yapılan allojeneik KHN uygun bir seçenektir. Özellikle refrakter hemolizi ve replasman ihtiyacı devam eden ve kemik iliği yetmezliği bulguları olan hasta grubunda allojeneik KHN bir tedavi seçeneği olarak değerlendirilmelidir.

## Introduction

Paroxysmal nocturnal hemoglobinuria (PNH) is an acquired clonal disorder characterized by a defect in the glycosylphosphatidyl-inositol (GPI) anchor protein, which results in partial or complete absence of GPI-linked proteins. In particular, the absence of GPI-linked CD55 and CD59 proteins leads to the increased sensitivity of erythrocytes to complement activation and complement-mediated hemolysis. The hallmarks of the disease are hemolysis, bone marrow failure, and increased risk of thromboembolism [1,2].

Eculizumab, a humanized monoclonal antibody that inhibits the formation of C5a and membrane attack complex, can control hemolysis effectively [3] and improve quality of life and survival rates while decreasing the incidence of thrombosis [3,4]. Although it has changed the fate of the disease, it does not cure it. Under these circumstances, it is an effective therapy for classical PNH (cPNH) patients with prominent intravascular hemolysis; however, its role in PNH with bone marrow failure is very limited. Furthermore, there is still a group of patients refractory to anticomplement therapy [5,6]. Considering all the available therapies, the only curative therapy is still allogeneic hematopoietic stem cell transplantation (allo-HCT), which is the effective and preferred therapy, especially in patients with bone marrow failure.

According to the literature, allo-HCT is generally limited to patients with incurable hemolysis under anticomplement therapy and patients with bone marrow failure due to a high risk of graft-versus-host disease (GVHD), transplant-related mortality and morbidity, and lack of suitable donors [7,8,9].

In this study, we retrospectively evaluate the efficiency and safety of allo-HCT in patients with cPNH and PNH with aplastic anemia (PNH-AA).

## Materials and Methods

### Patients

Thirty-five cPNH/PNH-AA patients who were treated with allo-HCT between October 2005 and April 2019 in 10 transplantation centers in Turkey were included. PNH was diagnosed by a flow cytometric method based on the analysis of CD55 and CD59 expression or by determining GPI-negative clones (granulocytes/monocytes) using fluorescent aerolysin (FLAER). cPNH was defined based on obvious hemolysis with abnormal lactate dehydrogenase (LDH) and other laboratory tests related to intravascular hemolysis. PNH-AA was defined in patients with evidence of AA with pancytopenia and related clinical presentation with a PNH clone with or without prominent hemolysis [5]. Patients with AA without PNH clones, patients with primary AA, patients with Fanconi anemia, and secondary AA cases other than PNH-AA were excluded. The indications for allo-HCT were incurable hemolysis with ongoing transfusion requirement and severe/very severe AA.

The study was approved by Ege University’s Ethics Committee and conducted in accordance with the Declaration of Helsinki.

### Transplantation

Human leukocyte antigen (HLA) matching for donor selection was based on serologic typing for HLA-A, -B, and -C antigens and on DNA typing for the HLA-DRB1 antigen. HLA-matched sibling donors (MSDs) were the first choice. When MSDs were unavailable, HLA-matched unrelated donors (MUDs) were preferred. Peripheral blood stem cells (PBSCs) were infused for 25 patients and bone marrow grafts were used for 10 patients.

### Conditioning Regimen

For 28 patients, a fludarabine-based reduced intensity chemotherapy (RIC) regimen was given, mainly cyclophosphamide, fludarabine, and antithymocyte globulin (ATG) or busulfan, fludarabine, and ATG. The patients were mostly treated with cyclosporine and methotrexate for GVHD prophylaxis according to the guidelines of the institution. For 7 patients, cyclophosphamide, busulfan and cyclophosphamide, and fludarabine regimens with or without ATG were used as myeloablative therapy. Cyclosporine and methotrexate were mainly used as GVHD prophylaxis. Data on the conditioning regimens and GVHD prophylaxis are provided in the Supplementary Table. Regarding the types of ATG, the rabbit type was used for all patients except 6 of them. The dose for rabbit ATG was 10-40 mg/kg/day for 2-4 days and the dose for the horse type was 2.5-5 mg/kg/day for 2-4 days according to the institutional protocol.

### Supportive Therapy

All patients were treated with antibacterial, antiviral, antifungal, and anti-*Pneumocystis jirovecii* infection prophylaxis with fluoroquinolones, acyclovir/valacyclovir, fluconazole, and trimethoprim/sulfamethoxazole, respectively.

Granulocyte colony-stimulating factor was started as indicated in the conditioning regimen protocol until myeloid recovery. Irradiated and leuko-depleted blood products were administered to maintain hemoglobin and platelet levels according to the threshold determined by the transplantation center. Ursodeoxycholic acid and defibrotide were administered for 8 and 3 patients, respectively, for prevention of veno-occlusive disease.

### Definitions

AA severity was defined according to the guidelines published by Marsh et al. [10]. Neutrophil and platelet engraftment were defined as an absolute neutrophil count (ANC) of >500/µL on 3 consecutive days and a platelet count of >20000/µL without transfusion support on 3 consecutive days, respectively. Primary graft failure was defined as failure to achieve engraftment 28 days after HCT. Secondary graft failure was defined as the development of ANC of <500/µL after achieving initial engraftment [11,12]. Acute and chronic GVHD were defined and graded according to the established guidelines [13,14]. GVHD-free, failure-free survival (GFFS) was reported as survival without grades 3-4 acute GVHD, moderate to severe chronic GVHD, or treatment failures, which were determined as death, primary or secondary graft failure, and relapse. Overall survival (OS) was defined as the time from transplantation until the time of death or the last follow-up.

### Statistical Analysis

Statistical analyses were performed using SPSS 16 (SPSS Inc., Chicago, IL, USA). The variables were first assessed by Kolmogorov-Smirnov/Shapiro-Wilk tests in terms of normal distribution. The results were provided as mean ± standard deviation for normally distributed variables and as median (minumum-maximum) for abnormally distributed parameters. All p-values were two-tailed and statistical significance was set at the level of p<0.05. Survival analysis was performed by log rank test and Kaplan-Meier curves. Values of p<0.05 were accepted as statistically significant.

## Results

### Patients

Thirty-five patients (19 male and 16 female) who were treated with allo-HCT in 10 transplantation centers were enrolled in this study. The median age at the time of transplantation was 32 years (minumum-maximum: 18-51). The characteristics of the patients are shown in Table 1.

Sixteen (45.7%) and 19 (54.3%) patients were diagnosed with cPNH and PNH-AA, respectively. The most common symptom displayed was fatigue, which was present in 25 (71.4%) patients, while dark urine was the least common symptom, present in only 2 (5.7%) patients. Dyspnea, abdominal pain, and jaundice were not reported in any cases. Six patients (17.1%, 5 patients with cPNH [31.2%] and 1 patient with PNH-AA [5.2%]) had a history of thromboembolism before transplantation, and 1 patient had multiple attacks. The sites for embolisms were the pulmonary vein, hepatic vein, and deep vein of the leg and upper extremities. No arterial embolisms were documented.

The therapies used before transplantation were steroids (12 patients), cyclosporine (13 patients), ATG (3 patients), growth factors and erythropoietin (2 patients), androgens (3 patients), and eculizumab (21 [60%] patients for a median of 24 months; minumum-maximum: 1-44). The patients were treated with a median of 2 different classes of therapies (minumum-maximum: 1-5) and the number of patients treated with more than 3 classes of therapies was 7 (20%). Fourteen (40%) patients were not treated with eculizumab because 9 patients were diagnosed before the availability of eculizumab in Turkey and 5 patients mainly presented with signs and symptoms of pancytopenia rather than hemolysis. Twenty-two patients were transfusion-dependent for both red blood cells and platelets. All patients were transfused with a median of 24 (minumum-maximum: 4-120) units of packed red blood cells and the median ferritin level before transplantation was 561 ng/mL (minumum-maximum: 18-5236). Ferritin values of ≥1500 mg/mL were only reported for 9 patients. Additionally, 14 patients were transfused with a median of 24 units (minumum-maximum: 4-74) of apheresis or pooled platelets. The PRBC dependence did not differ between patients with cPNH and PNH-AA (p>0.05).

### Transplantation

The median time to transplantation was 36 months (minumum-maximum: 3-240). The median hemoglobin (Hb), leukocyte, neutrophil, platelet, and LDH levels were 6.8 g/dL (minumum-maximum: 5-9.8), 3360/µL (minumum-maximum: 100-13500), 680/µL (minumum-maximum: 90-13200), 56000/µL (minumum-maximum: 6000-340000), and 882 IU/L (minumum-maximum: 230-4440), respectively. The median neutrophil count (1800/µL versus 450/µL, p=0.001), leukocyte count (4000/µL versus 1700/µL, p=0.004), platelet count (125000/µL versus 18000/µL, p=0.001), LDH (1144 IU/L versus 310 IU/L, p=0.016), and bone marrow cellularity (60% versus 15%, p<0.0001) were statistically different between patients with cPNH and PNH-AA. The laboratory parameters and characteristics of the patients are shown in Table 1.

The median percentages of PNH clone in granulocytes and monocytes before transplantation were 62.7% (2.6%-97%) and 60% (3.1%-95%), respectively. PNH clone size for granulocytes was statistically different between cPNH and PNH-AA patients (90% versus 30%, p=0.04). Allo-HCT was performed with MUDs and MSDs for 12 and 23 patients, respectively. In the subgroup analysis of patients with cPNH and PNH-AA, transplantation from a MSD was much more frequent in patients with cPNH (p=0.039).

Myeloablative regimens and RIC protocols were used for 7 (20%) and 28 (80%) patients, respectively. A bone marrow source was used for 10 patients, and PBSCs were transfused for 25 patients. The median number of stem cells infused was 2.54x10^6^ (2.17-3.91x10^6^) and 7x10^6^ (3.39-11.3x10^6^) CD34+ cells/kg recipient weight for bone marrow harvesting and PBSCs, respectively.

Platelet and neutrophil engraftments were achieved in 28 (80%) and 29 (82.8%) cases, and primary graft failure was documented in 6 (17.1%) cases. Graft failure rates did not differ between patients with cPNH and PNH-AA. The patients with primary graft failure died due to refractory disease, pancytopenia, and sepsis. Secondary graft failure was reported in only 1 patient who died due to aplastic bone marrow, sepsis, and peripheral lymphoproliferative disease. The median days to neutrophil and platelet engraftment were 16 (10-27) and 16 (11-27) days, respectively. The median days to both neutrophil and platelet engraftments did not differ between the patients with cPNH and PNH-AA or patients receiving transplants from MUDs and MSDs.

Six (17.1%) patients had acute GVHD, including cutaneous in 5 patients, gastrointestinal system in 1 patient, and liver in 1 patient. All cases of acute GVHD were grade 1 or 2. RIC and cyclosporine/methotrexate regimens were given to all patients with acute GVHD for conditioning and GVHD prophylaxis, respectively. There was no reported acute GVHD-related death. Four patients (11.4%) had mild chronic GVHD, affecting the eyes (3 patients), liver (2 patients), and skin (1 patient). All patients with chronic GVHD were treated with RIC protocols as the conditioning regimen, and 3 of 4 patients received PBSCs. All patients documented with chronic GVHD were alive at the last follow-up visit and the GVHD was under control with immunosuppressive therapy given according to the guidelines of the center. Presence of chronic or acute GVHD did not differ between the patients with cPNH and PNH-AA or the patients with MUDs and MSDs.

Possible and probable fungal pneumonia and CMV reactivation were both documented in 4 patients. Two of these patients experienced both CMV activation and possible fungal pneumonia.

### Survival

The median follow-up duration was 36 months (minumum-maximum: 2-156). The PNH clone was detectable in only 4 patients at the last visit. The percentages of the clone in monocytes were 0.3%, 1%, 1.2%, and 2% and in granulocytes were 0.2%, 2%, 2%, and 3%. The chimerism levels were 98%, 93%, 96%, and 98%, respectively, at the last visit. All patients with detectable PNH clones were alive at the last visit and were transfusion-independent.

Six patients died during the follow-up period. For 5 patients, graft failure and related pancytopenia and sepsis were the causes of death; 1 patient with secondary graft failure died due to peripheral T-cell and lymphoproliferative disease and sepsis. All patients were treated with RIC protocols, except 1 patient who had a myeloablative conditioning regimen. Transplantations from MUDs and MSDs were performed for 2 and 3 patients, respectively.

**Figure 1:**
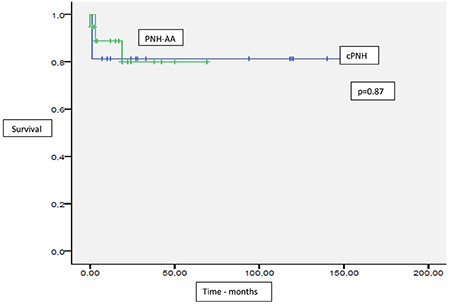
Overall survival in paroxysmal nocturnal hemoglobinuria (PNH) patients with or without anaplastic anemia. cPNH: Classical PNH; PNH-AA: PNH with aplastic anemia.

**Figure 2:**
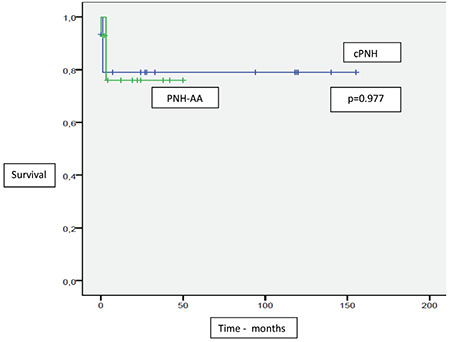
Graft-versus-host disease-free, failure-free survival in paroxysmal nocturnal hemoglobinuria (PNH) patients with or without anaplastic anemia. cPNH: Classical PNH; PNH-AA: PNH with aplastic anemia.

Two-year OS and GFFS were 81.2% and 78.1%, respectively, and mean OS and GFFS were 126.9±10.4 months and 121.4±12.1 months, respectively. The 2-year OS for cPNH and PNH-AA was 81.3% and 79.9%, respectively (p=0.87). The 2-year GFFS for cPNH and PNH-AA was 79% and 76%. There was no statistical difference between the two groups (p=0.977) (Figures 1 and 2). In patients receiving transplantations from MSDs and MUDs, although 2-year OS and GFFS rates were higher in patients with MSDs, this difference did not reach statistical significance (2-year OS: 83.3% versus 69% [p=0.44] for MSDs and MUDs, respectively; GFFS: 83% versus 70.7% [p=0.62] for MSDs and MUDs, respectively). Although there was a difference between the groups, this difference did not reach statistical significance, probably due to the small size of the cohort.

## Discussion

Although anticomplement therapy with eculizumab is very effective in cPNH patients, refractoriness is a problem. Furthermore, eculizumab is not an option for patients with bone marrow failure without obvious hemolysis. Under these circumstances, allo-HCT is an option for patients with ongoing hemolysis and PNH-AA.

In this multicenter study, we retrospectively evaluated the allo-HCT results of 35 patients with cPNH and PNH-AA. The median age at the time of transplantation was 32 and the oldest patient was 51 years of age. In the literature, the median ages of patients were generally in the range of 24.5-37 years, but a patient as old as 60 years was transplanted [11,12,15,16,17,18]. The median time to transplantation was 36 months in our cohort, and patients received different therapies, including eculizumab (21 patients with a median of 24 months; minumum-maximum: 1-44 months). In the literature, the median follow-up period before transplantation was 6-28 months [11,12,19,20]. The median time to transplantation was reported to be as short as 6 months by Liu et al. [11], probably due to the unavailability of eculizumab in China. In the present study, time to transplantation was relatively long because patients were treated with different modalities, including eculizumab. Additionally, searching for a suitable related or unrelated donor might also increase the time to transplantation.

Laboratory parameters are summarized in Table 1. The median neutrophil count, leukocyte count, platelet count, LDH level, and bone marrow cellularity were statistically different in patients with cPNH and PNH-AA. Although we did not find a correlation between survival and laboratory parameters, Bemba et al. [20] reported better survival rates in patients with ANC of >1000/µL and hemoglobin of >9 g/dL at transplantation; however, this finding was not confirmed by other studies. The median PNH clone size for granulocytes in cPNH cases was higher than in PNH-AA (90% in cPNH versus 30% in PNH-AA [p=0.04]), which was compatible with the literature [12]. Neither our study nor other studies have documented a survival benefit related to PNH clone size.

Primary engraftment failure was reported in 6 (17.1%) patients and secondary failure was observed in only 1 patient. The median time to neutrophil and platelet engraftment was 16 (10-27) and 16 (11-27) days, respectively. The primary graft failure rate was higher in our study, mainly due to differences in definitions. In the study conducted by Liu et al. [11], there was no reported primary graft failure; however, the range of time to platelet engraftment was up to 75 days, and there were 3 patients (6.82%) with delayed platelet engraftment. The median time to neutrophil and platelet engraftment in our cohort was within the range reported in the literature (13-20.5 days for neutrophils and 12-27 for platelets) [15,16,18,21].

In our study, the rates of 2-year OS and GFFS were 81.2% and 78.1%, respectively, and the mean OS and GFFS were 126.9±10.4 months and 121.4±12.1 months, respectively. Recently, a study by Pantin et al. [16] analyzed the long-term outcome of fludarabine-based RIC allo-HCT in 17 patients with cPNH and PNH-AA. The conditioning and GVHD prophylaxis regimens were similar to those of our study. With a follow-up of a median of nearly 6 years, 87.8% patients survived. The survivors were transfusion-independent and there was no evidence of PNH [16]. Another retrospective study evaluating RIC regimens in 33 PNH patients with or without AA also demonstrated excellent survival rates. At a median follow-up of 57.0 months (minumum-maximum: 6.0-151.3), the estimated OS was 87.9±5.7% [12]. The low transfusion rates and transplantation with the RIC regimen were probably related to the higher survival rates.

Beside these studies, data from previous studies also revealed poor survival rates with allo-HCT [19,20]. Saso et al. [19] revealed the results of allo-HCT performed between 1978 and 1995. In this study, the 2-year probability of survival was as low as 56%, and transplantation could restore normal marrow function in only about 50% of patients. However, in this study, there were several factors that might have been related to the lower survival rates. First of all, the patients were heavily transfused, and platelet refractoriness was documented in more than 20% of cases. Secondly, myeloablative regimens were mostly used, and the rates of acute and chronic GVHD (34% and 33%, respectively) were high. These characteristics of the patients might be related to lower survival rates compared to the results in our study.

Considering the rarity of this disease and other treatment options, although prospective randomized studies are lacking in the literature, the safety and efficiency of RIC regimens were demonstrated in small series [17,22]. By integrating RIC regimens with allo-HCT for PNH patients, the mortality and morbidity rates have been reduced, obviating the need for more potentially toxic myeloablative therapies [16,23].

In our cohort, only 4 of the patients had a detectable PNH clone in a range of 0.2%-3%. All patients with detectable clones were transfusion-independent. In 88.5% of the patients, the PNH clone was undetectable at the last follow-up. PNH clone negativity was also high after allo-HCT in other studies [16,18].

When we performed a subgroup analysis including cPNH and PNH-AA, the rates of 2-year OS (81.3% in cPNH and 79.9% in PNH-AA, p=0.87) and GFFS (79% in cPNH and 76% in PNH-AA, p=0.977) were not statistically different. Similar to our results, Liu et al. [11] reported that 3-year OS and GFFS were 90.4% and 85.6%, respectively, without showing a statistical difference. Although GFFS tended to be better for cPNH patients in both our study and the study by Liu et al. [11], this difference did not reach statistical significance.

In addition, we did not discover a statistically significant difference between transplantation from MUDs or MSDs. Although data from previous studies reported that MUD allografts might result in high mortality rates [19], other studies documented successful transplantation results with MUD allografts [24,25,26]. In patients without suitable sibling donors, unrelated donors can be a particularly good option.

In terms of GVHD, we documented acute GVHD and chronic GVHD in 17.1% and 11.4% of the patients, respectively. Thus, we have reported relatively low GVHD rates. This might be due to several reasons. First of all, this was a retrospective study and loss of data is one of the major limitations in retrospective studies. Secondly, a regimen including ATG was used for all patients except 2 of them. It is well known that regimens with ATG are associated with low GVHD rates. Another reason for low GVHD might be the wide range of follow-up time. Although the median follow-up duration was 36 months, the range was between 2 and 156 months. Patients with short follow-up periods might decrease the overall GVHD rate. A very recent study including PNH patients with allogeneic transplantation reported the rate of acute GVHD at 100 days as 21%. The cumulative incidence of extensive chronic GVHD at 2 years was 17% [27]. The Polish Adult Leukemia Group also reported acute GVHD and cumulative 1-year incidence of extensive chronic GVHD as 23% and 10.8%, respectively [28].

### Study Limitations

Our study had some limitations. First of all, it was a retrospective study; therefore, missing data and loss of follow-up visits constituted a problem. Although it was a multicenter study including a relatively large cohort, it only included matched related and unrelated donors. There were no data for haploidentical transplantation in PNH patients. In spite of these limitations, this is the first multicenter study to report allo-HCT results from Turkey; considering the rarity of this disease, our study included a relatively large cohort with 35 patients.

## Conclusion

Allo-HCT with MSDs and MUDs is a good option for selected patients with cPNH and PNH-AA. Patients with debilitating and refractory hemolysis and patients with bone marrow failure might form a particularly excellent group of candidates for allo-HCT. In the era of complement inhibitors, identifying the patients who will benefit from allo-HCT is a very important issue. Even though allo-HCT is the only curative therapy, its mortality and morbidity rates remain an issue. Although there are still unanswered questions, allo-HCT is an option for selected patients with PNH.

## Figures and Tables

**Table 1:**
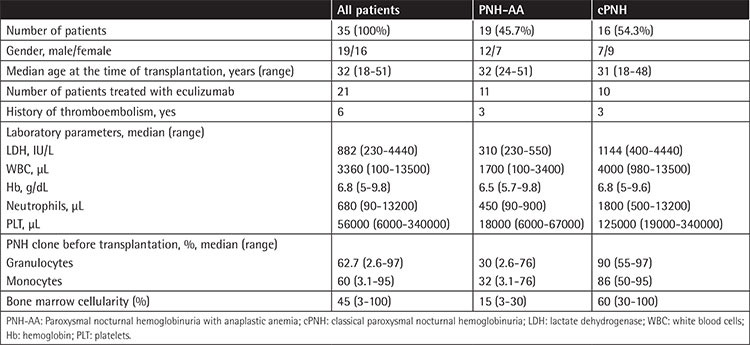
Characteristics of the patients.

**Supplementary Table:**
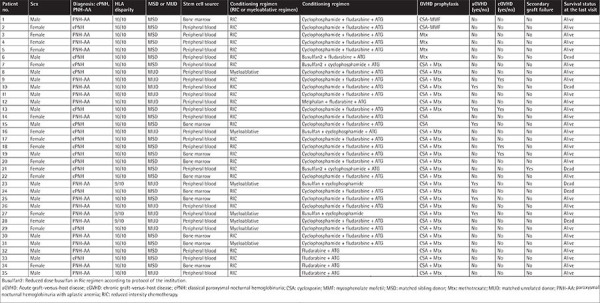
Transplant data of the patients.
